# 2-[3-(1*H*-Benzimidazol-2-yl)prop­yl]-1*H*-benzimidazol-3-ium 3,4,5-tri­hydroxy­benzoate trihydrate

**DOI:** 10.1107/S2414314625002561

**Published:** 2025-03-27

**Authors:** José Carlos Palacios Rodríguez, Angel Mendoza, Martha Sosa Rivadeneyra, Sylvain Bernès

**Affiliations:** a Facultad de Ciencias Químicas, Benemérita Universidad Autónoma de Puebla, 72570 Puebla, Pue., Mexico; b Instituto de Ciencias, Benemérita Universidad Autónoma de Puebla, 72570 Puebla, Pue., Mexico; c Instituto de Física, Benemérita Universidad Autónoma de Puebla, 72570 Puebla, Pue., Mexico; Goethe-Universität Frankfurt, Germany

**Keywords:** crystal structure, benzimidazole, gallic acid, hydrate, hydrogen bonds

## Abstract

The characterized organic trihydrate title salt, C_17_H_17_N_4_^+^·C_7_H_5_O_5_^−^·3H_2_O or (H*L*)^+^(Gal)^−^·3H_2_O, with *L* = 1,3-bis­(1*H*-benzimidazol-2-yl)propane (C_17_H_16_N_4_) and HGal = 3,4,5-tri­hydroxy­benzoic acid (gallic acid, C_7_H_6_O_5_), crystallizes with two formulas per asymmetric unit. One-half of the cations in the crystal feature a bent shape, with the central propyl chain having a *gauche* conformation, and the other half is nearly linear, with a *trans* propyl chain. Both cations form two independent herringbone layers in the crystal, which allow efficient π–π inter­actions between aromatic rings of the benzimidazole moieties. These layers are parallel to (100), and anions and water mol­ecules of crystallization inter­calate between these cationic planes. All potential donor groups for hydrogen bonding (NH and OH groups) actually form hydrogen bonds, ensuring a good cohesion between layers of cations and anions, stacked along [100].

## Abstract 2

A layered crystal structure built up from alternating cationic and (anionic + water of crystallization) supra­molecular planes parallel to (100) is described for the title hydrated salt.

## Graphical abstract

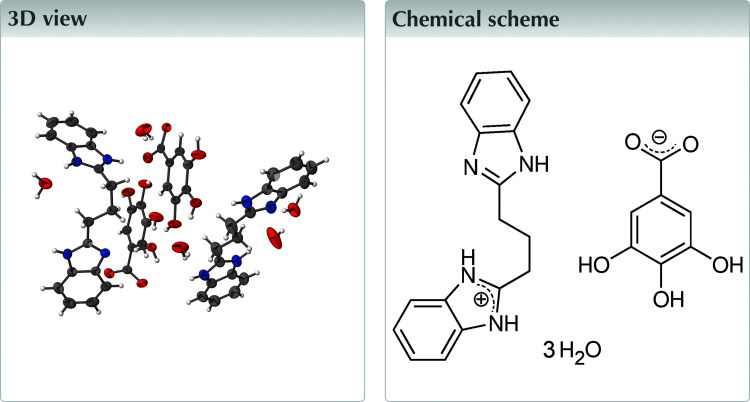

## Structure description

The title salt was isolated during an exploratory synthetic effort aiming to describe the ability of gallic acid (3,4,5-tri­hydroxy­benzoic acid, HGal) to co-crystallize with imidazole, benzimidazole derivatives and related bases. According to the Δp*K*_a_ rule, the formation of true cocrystals rather than salts is quite unpredictable for such acid–base pairs: with p*K*_a_ ≃ 5.3 for the conjugate acid of benzimidazole and p*K*_a_ ≃ 4.5 for gallic acid, Δp*K*_a_ ≃ 0.8 falls in the grey zone of the Δp*K*_a_ rule (Cruz-Cabeza, 2012 ▸). Indeed cocrystals based on HGal were reported, for example with metronidazole (Zheng *et al.*, 2019 ▸) or penciclovir (Yuan *et al.*, 2020 ▸), while a gallate salt was obtained with 2-methyl-benzimidazole (Sosa-Rivadeneyra *et al.*, 2024 ▸). We also reported recently the structure of a salt cocrystal in which HGal partially transfers protons to a bis-benzimidazole compound (Palacios Rodríguez *et al.*, 2023 ▸). The herein reported structure is closely related to this salt cocrystal, as it represents the salt part of the salt cocrystal.

**Figure 1:**
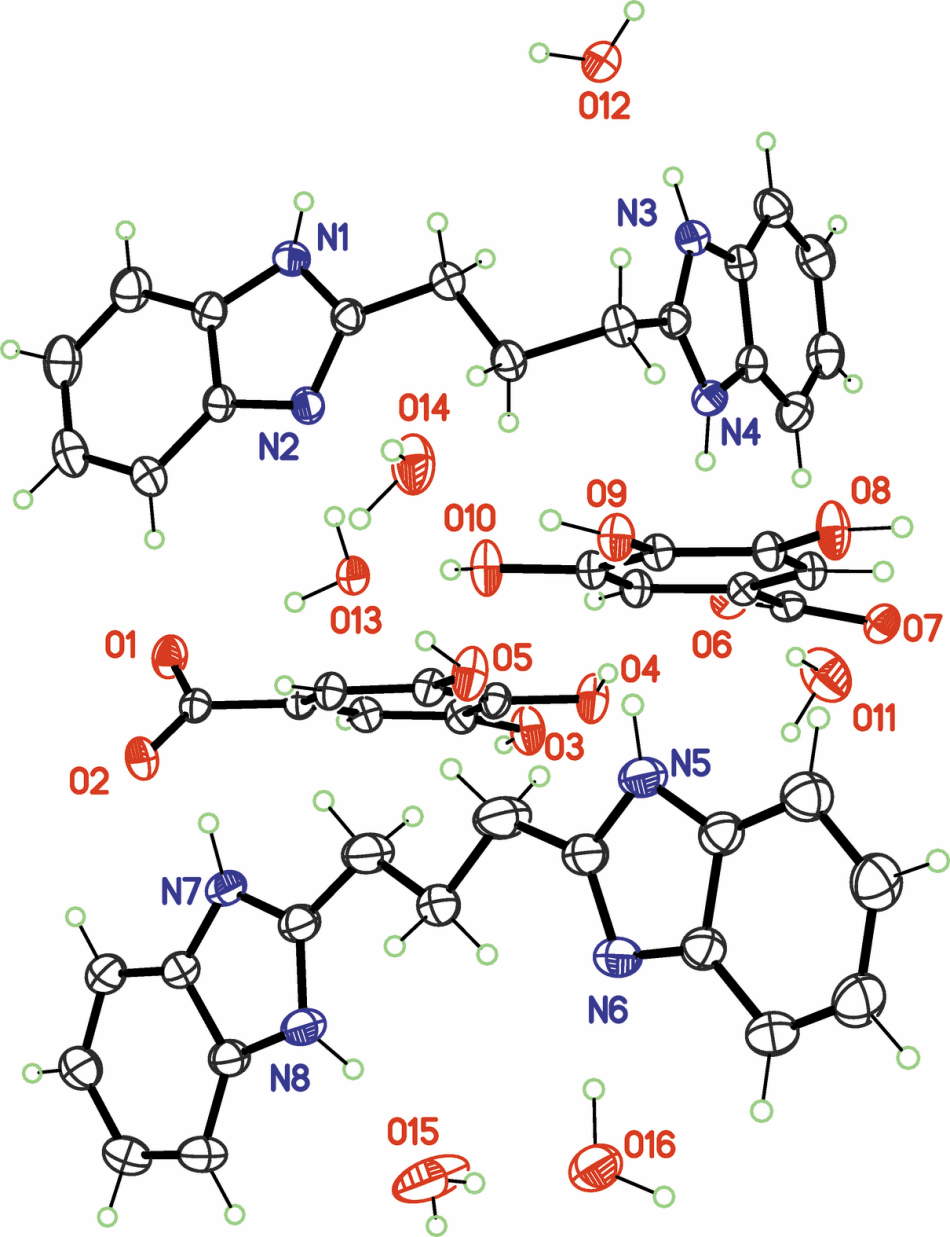
The mol­ecular structure (asymmetric unit), with displacement ellipsoids at the 30% probability level.

The chemical formula of the title compound is (H*L*)^+^(Gal)^−^·3H_2_O where *L* is 1,3-bis­(1*H*-benzimidazol-2-yl)propane. The asymmetric unit contains twice this formula (*Z*′ = 2; Fig. 1 ▸), with all mol­ecules placed in general positions. This is probably a consequence of the stabilization of two conformers for the cations H*L^+^*. The first independent cation, C1–C17, displays an angular shape, with the central propyl chain having a *gauche* conformation [C2—C3—C4—C5 = −69.39 (16)°]. The dihedral angle between benzimidazole rings in this cation is 86.50 (2)°. In contrast, the other cation, C18—C34, is nearly planar, with a *trans* propyl chain [C19—C20—C21—C22 = −177.07 (19)°], and a dihedral angle of 4.55 (6)° between benzimidazole rings. Gallate ions also display different conformations, mainly for the carb­oxy­lic group, which is twisted by 14.34 (4) or 48.75 (5)° with respect to the benzene ring.

**Figure 2:**
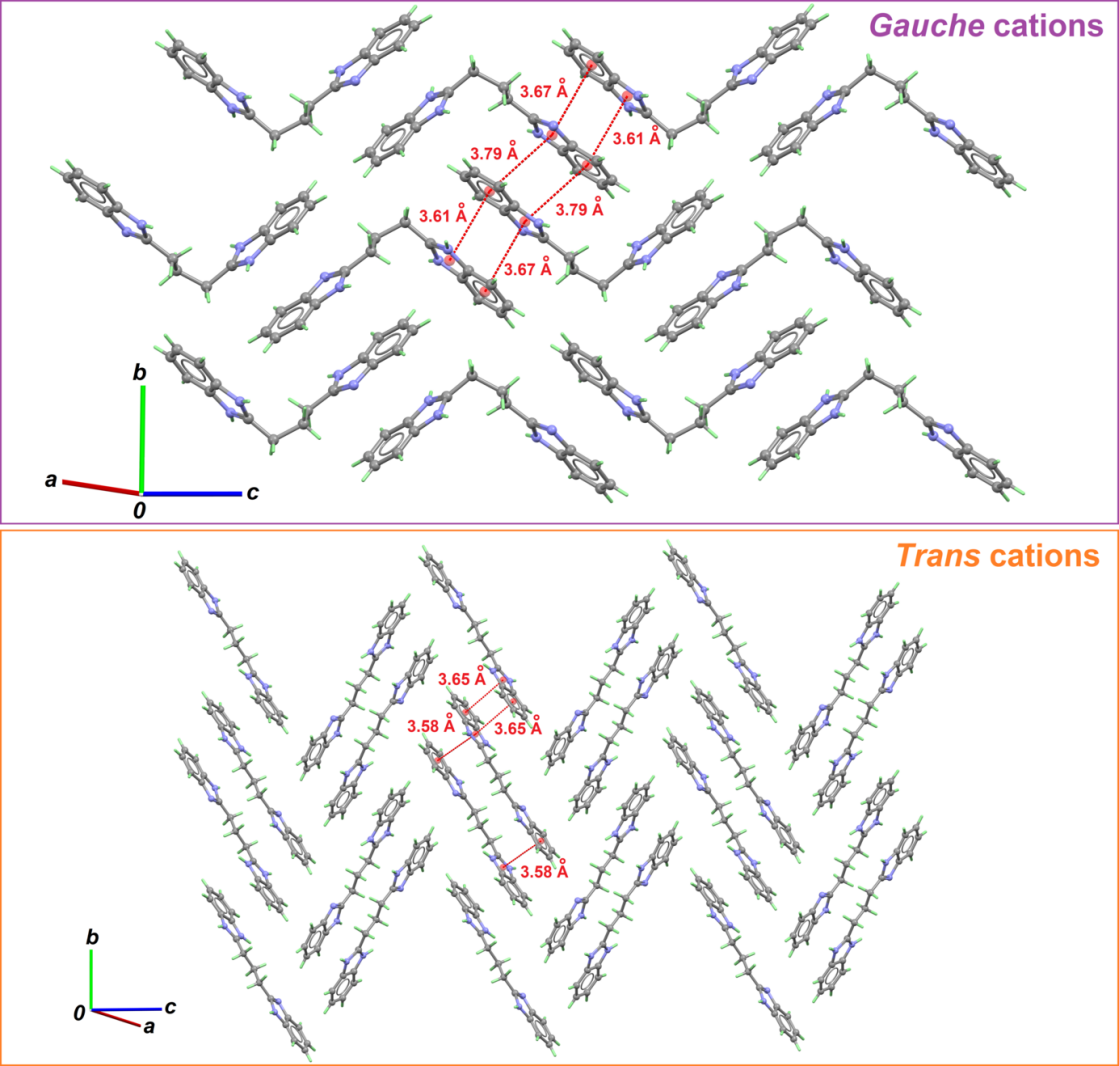
Herringbone arrangements observed for H*L^+^* cations in the crystal, with shortest π–π separations between aromatic rings. The top panel is for cations having a bent shape, due to the *gauche* conformation of the central propyl chain, and the bottom panel is for linear cations featuring a *trans* propyl chain. Both projections are nearly normal to [100].

**Figure 3:**
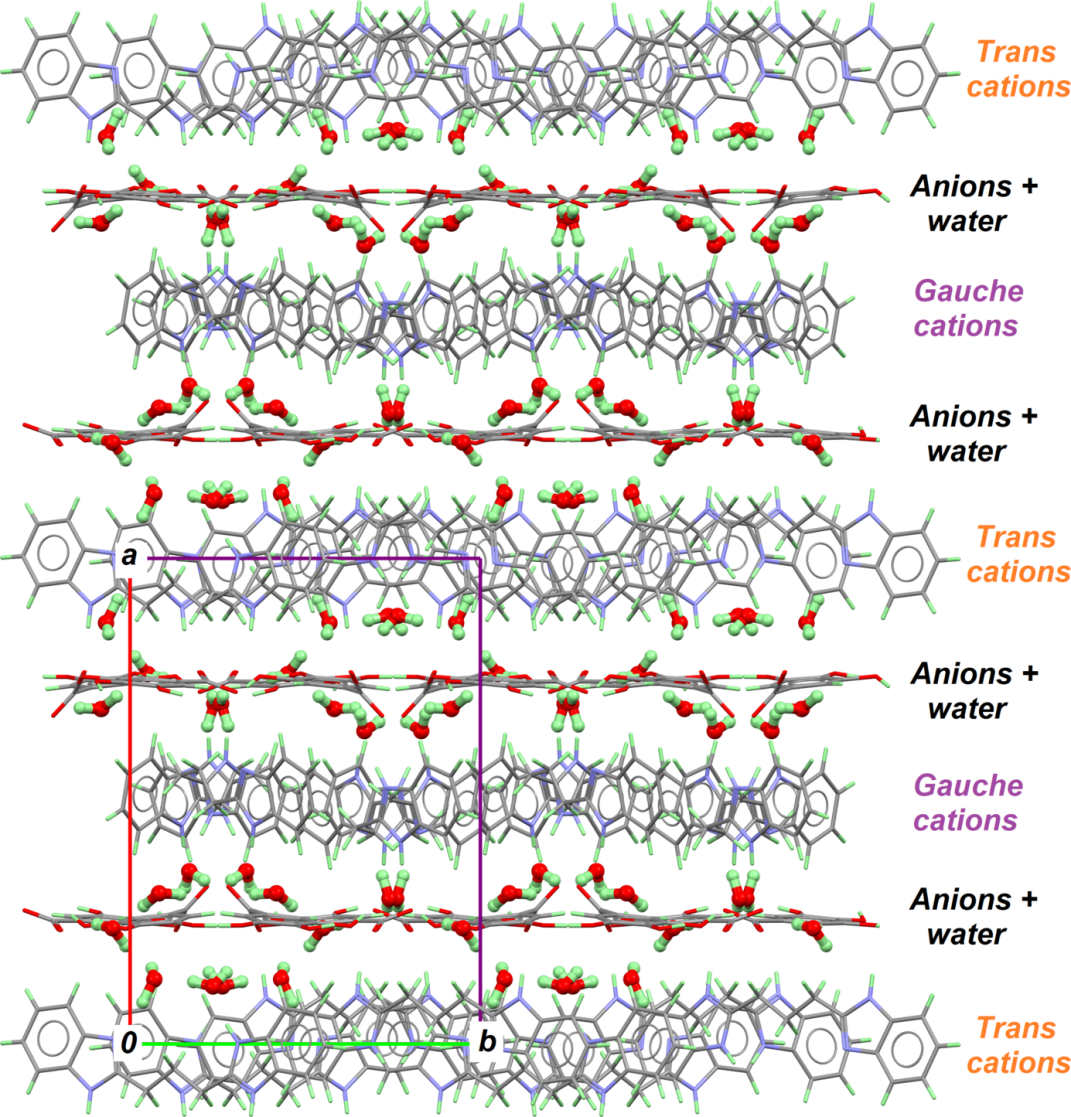
The crystal packing, as viewed down crystallographic *c* axis, emphasizing the layered structure, with alternating cationic and anionic planes. Water mol­ecules are shown using a ball-and-stick representation to emphasize their positions between cationic planes.

Both cations are arranged in such a way that independent two-dimensional patterns are formed, favouring π–π contacts. *Gauche* cations form a herringbone pattern, characterized by aromatic rings giving face-to-face inter­actions with separations of 3.608 (1) and 3.674 (1) Å (Fig. 2 ▸, top). These cations are segregated in planes parallel to (100). Another herringbone plane is formed by *trans*-conformed cations, which is parallel to the previous one, and displays a more acute stepper angle (Fig. 2 ▸, bottom). In this plane, short π–π contacts range from 3.580 (1) to 3.646 (1) Å. Gallate anions and water mol­ecules are sandwiched between *gauche*- and *trans*-cation layers (Fig. 3 ▸). The resulting crystal structure, based on charged supra­molecular planes stacked along the largest unit cell axis, is entirely different from that observed for (H*L*^+^·Gal^−^)_2_·*L*·(ethyl acetate)_2.94_, in which the supra­molecular structure is cylindrical and no π–π contacts stabilize the structure (Palacios Rodríguez *et al.*, 2023 ▸). This could be a direct consequence of the solvent used for crystallization: (*HL*^+^·Gal^−^)_2_·*L*·(ethyl acetate)_2.94_ was crystallized from ethyl acetate, a poor donor/acceptor for hydrogen bonding, while the title compound (H*L*)^+^(Gal)^−^·3H_2_O was obtained from a methanol solution. The insertion a water mol­ecules in the structure is attributed to the fact that non-dried methanol was used. Moreover, with such non-controlled experimental conditions, it has been reported that the formation of pharmaceutical cocrystal hydrates can be obtained under conditions of high relative humidity (Karki *et al.*, 2007 ▸).

The presence of H_2_O in the herein reported structure is essential for crystal cohesion. Indeed, all NH and OH groups in the crystal behave as donors for hydrogen bonding, forming an extensive three-dimensional network of hydrogen bonds (Table 1 ▸). Almost all hydrogen bonds are significant in terms of stabilization energy: 22 of 24 contacts have a *D*—H⋯*A* angle greater than 150°, and H⋯*A* separations range from 1.71 (2) to 2.330 (18) Å. According to the ‘graph-sets’ tool available in *Mercury* (Macrae *et al.*, 2020 ▸), all ring motifs are of level 3 (or higher) and include between three and ten mol­ecules. The smallest motif, 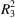
(12), involves one cation, one anion and one water mol­ecule, and rings as large as 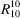
(68) are formed, involving three neighbouring supra­molecular layers in the crystal.

## Synthesis and crystallization

A solution of 1,3-bis­(1*H*-benzo[*d*]imidazol-2-yl)propane (*L*, 12.4 mg, 0.045 mmol) and gallic acid (HGal, 7.6 mg, 0.045 mmol) in 10 ml of methanol was heated at boiling temperature until dissolution of the reactants. After filtration, the solution was left at room temperature for slow evaporation of the solvent, giving brown crystals suitable for single-crystal X-ray diffraction analysis.

## Refinement

Crystal data, data collection and structure refinement details are summarized in Table 2 ▸.

## Supplementary Material

Crystal structure: contains datablock(s) I, global. DOI: 10.1107/S2414314625002561/bt4166sup1.cif

Structure factors: contains datablock(s) I. DOI: 10.1107/S2414314625002561/bt4166Isup2.hkl

Supporting information file. DOI: 10.1107/S2414314625002561/bt4166Isup3.cml

CCDC reference: 2432736

Additional supporting information:  crystallographic information; 3D view; checkCIF report

## Figures and Tables

**Table 1:** Hydrogen-bond geometry (Å, °)

*D*—H⋯*A*	*D*—H	H⋯*A*	*D*⋯*A*	*D*—H⋯*A*
N1—H1⋯O1^i^	0.818 (18)	2.330 (18)	3.0706 (17)	150.9 (16)
N3—H3⋯O12	0.885 (17)	1.830 (18)	2.6968 (17)	166.0 (16)
N4—H4⋯O6	0.878 (17)	1.884 (18)	2.7290 (15)	160.9 (16)
N5—H5⋯O9	0.87 (2)	2.18 (2)	3.0248 (17)	162.9 (18)
N7—H7*A*⋯O7^ii^	0.879 (19)	1.84 (2)	2.7045 (15)	165.7 (18)
N8—H8*A*⋯O15	0.919 (19)	1.78 (2)	2.676 (2)	165.3 (18)
O3—H3*C*⋯O8^iii^	0.90 (2)	1.95 (2)	2.7201 (14)	142.6 (19)
O4—H4*C*⋯O6	0.93 (2)	2.18 (2)	2.9509 (14)	139.8 (19)
O5—H5*A*⋯O13	0.92 (2)	1.71 (2)	2.6263 (14)	173 (2)
O8—H8*B*⋯O2^iv^	0.87 (2)	1.75 (2)	2.6171 (13)	174 (2)
O9—H9*A*⋯O14	0.82 (2)	1.81 (2)	2.6193 (15)	168.4 (19)
O10—H10*A*⋯O13	0.86 (2)	1.83 (2)	2.6808 (13)	168 (2)
O11—H11*A*⋯O6	0.88 (3)	2.04 (3)	2.9148 (17)	174 (3)
O11—H11*B*⋯O16^v^	0.96 (3)	1.91 (3)	2.865 (2)	175 (3)
O12—H12*A*⋯O1^i^	0.95 (3)	1.86 (3)	2.7796 (17)	162 (2)
O12—H12*B*⋯O3^vi^	0.83 (3)	2.14 (3)	2.9254 (18)	158 (2)
O13—H13*A*⋯N2	0.923 (19)	1.81 (2)	2.7348 (15)	175.0 (17)
O13—H13*B*⋯O7^ii^	0.856 (19)	1.89 (2)	2.7249 (13)	165.5 (18)
O14—H14*A*⋯O1^vii^	0.94 (3)	1.96 (3)	2.8608 (18)	160 (2)
O14—H14*B*⋯O11^ii^	0.97 (3)	1.85 (3)	2.814 (2)	177 (2)
O15—H15*A*⋯O2^viii^	0.77 (5)	2.10 (5)	2.865 (2)	171 (5)
O15—H15*B*⋯O16	0.86 (5)	1.91 (5)	2.742 (2)	165 (4)
O16—H16*A*⋯O4^ix^	0.91 (3)	2.22 (3)	3.0444 (18)	150 (2)
O16—H16*B*⋯N6	1.01 (3)	1.80 (3)	2.800 (2)	167 (2)

Symmetry codes: (i) 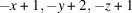; (ii) 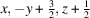; (iii) 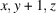; (iv) 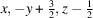; (v) 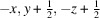; (vi) 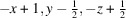; (vii) 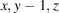; (viii) 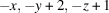; (ix) 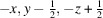.

**Table 2:** Experimental details

Crystal data	
Chemical formula	C_17_H_17_N_4_^+^·C_7_H_5_O_5_^−^·3H_2_O
*M*_r_	500.50
Crystal system, space group	Monoclinic, *P*2_1_/*c*
Temperature (K)	296
*a*, *b*, *c* (Å)	19.1096 (3), 13.69762 (18), 18.5399 (2)
β (°)	96.4031 (12)
*V* (Å^3^)	4822.66 (11)
*Z*	8
Radiation type	Mo *K*α
μ (mm^−1^)	0.11
Crystal size (mm)	0.66 × 0.49 × 0.12

Data collection	
Diffractometer	Xcalibur, Atlas, Gemini
Absorption correction	Multi-scan (*CrysAlis PRO*; Rigaku OD, 2022 ▸)
*T*_min_, *T*_max_	0.906, 1.000
No. of measured, independent and observed [*I* > 2σ(*I*)] reflections	115888, 14720, 10544
*R*_int_	0.077
(sin θ/λ)_max_ (Å^−1^)	0.714

Refinement	
*R*[*F*^2^ > 2σ(*F*^2^)], *wR*(*F*^2^), *S*	0.049, 0.145, 1.04
No. of reflections	14720
No. of parameters	722
H-atom treatment	H atoms treated by a mixture of independent and constrained refinement
Δρ_max_, Δρ_min_ (e Å^−3^)	0.54, −0.29

Computer programs: *CrysAlis PRO* (Rigaku OD, 2022 ▸), *SHELXT2018/2* (Sheldrick, 2015*a* ▸), *SHELXL2019/3* (Sheldrick, 2015*b* ▸), *XP* in *SHELXTL-Plus* (Sheldrick, 2008 ▸), *Mercury* (Macrae *et al.*, 2020 ▸) and *publCIF* (Westrip, 2010 ▸).

## full crystallographic data

### 2-[3-(1H-Benzimidazol-2-yl)propyl]-1H-benzimidazol-3-ium 3,4,5-trihydroxybenzoate trihydrate Crystal data

**Table:**

C_17_H_17_N_4_^+^·C_7_H_5_O_5_^−^·3H_2_O	*F*(000) = 2112
*M**_r_* = 500.50	*D*_x_ = 1.379 Mg m^−^^3^
Monoclinic, *P*2_1_/*c*	Mo *K*α radiation, λ = 0.71073 Å
*a* = 19.1096 (3) Å	Cell parameters from 34690 reflections
*b* = 13.69762 (18) Å	θ = 1.8–32.0°
*c* = 18.5399 (2) Å	µ = 0.11 mm^−^^1^
β = 96.4031 (12)°	*T* = 296 K
*V* = 4822.66 (11) Å^3^	Trapezoid, brown
*Z* = 8	0.66 × 0.49 × 0.12 mm

### 2-[3-(1H-Benzimidazol-2-yl)propyl]-1H-benzimidazol-3-ium 3,4,5-trihydroxybenzoate trihydrate Data collection

**Table:**

Xcalibur, Atlas, Gemini diffractometer	14720 independent reflections
Radiation source: Sealed X-ray tube	10544 reflections with *I* > 2σ(*I*)
Detector resolution: 10.5564 pixels mm^-1^	*R*_int_ = 0.077
ω scans	θ_max_ = 30.5°, θ_min_ = 1.8°
Absorption correction: multi-scan (CrysAlisPro; Rigaku OD, 2022)	*h* = −27→27
*T*_min_ = 0.906, *T*_max_ = 1.000	*k* = −19→19
115888 measured reflections	*l* = −26→26

### 2-[3-(1H-Benzimidazol-2-yl)propyl]-1H-benzimidazol-3-ium 3,4,5-trihydroxybenzoate trihydrate Refinement

**Table:**

Refinement on *F*^2^	Secondary atom site location: difference Fourier map
Least-squares matrix: full	Hydrogen site location: mixed
*R*[*F*^2^ > 2σ(*F*^2^)] = 0.049	H atoms treated by a mixture of independent and constrained refinement
*wR*(*F*^2^) = 0.145	*w* = 1/[σ^2^(*F*_o_^2^) + (0.0739*P*)^2^ + 0.6719*P*] where *P* = (*F*_o_^2^ + 2*F*_c_^2^)/3
*S* = 1.04	(Δ/σ)_max_ = 0.001
14720 reflections	Δρ_max_ = 0.54 e Å^−^^3^
722 parameters	Δρ_min_ = −0.29 e Å^−^^3^
0 restraints	Extinction correction: SHELXL-2019/3 (Sheldrick, 2015b), Fc^*^=kFc[1+0.001xFc^2^λ^3^/sin(2θ)]^-1/4^
0 constraints	Extinction coefficient: 0.00053 (19)
Primary atom site location: dual	

### 2-[3-(1H-Benzimidazol-2-yl)propyl]-1H-benzimidazol-3-ium 3,4,5-trihydroxybenzoate trihydrate Special details

**Table:**

Refinement. All O- and N-bonded H atoms were found in difference maps and refined with free coordinates, while C-bonded H atoms were placed in calculated positions. H atoms were refined with calculated isotropic displacement parameters, using *U*_iso_(H) = *xU*_eq_(parent atoms), *x* = 1.5 if the parent atom is O, and *x* = 1.2 otherwise. No geometric restraints were applied, in order to obtain unbiased dimensions for hydrogen bonds.

### 2-[3-(1H-Benzimidazol-2-yl)propyl]-1H-benzimidazol-3-ium 3,4,5-trihydroxybenzoate trihydrate Fractional atomic coordinates and isotropic or equivalent isotropic displacement parameters (Å^2^)

**Table:**

	*x*	*y*	*z*	*U*_iso_*/*U*_eq_	
N1	0.55021 (6)	0.85336 (9)	0.51878 (6)	0.0410 (3)	
H1	0.5912 (10)	0.8474 (13)	0.5106 (10)	0.049*	
N2	0.43524 (6)	0.82506 (8)	0.51117 (6)	0.0372 (2)	
N3	0.52713 (6)	0.73150 (9)	0.23807 (6)	0.0376 (2)	
H3	0.5667 (9)	0.7051 (12)	0.2585 (9)	0.045*	
N4	0.41720 (6)	0.77296 (8)	0.21795 (6)	0.0369 (2)	
H4	0.3718 (9)	0.7759 (12)	0.2215 (9)	0.044*	
C1	0.49459 (7)	0.80468 (10)	0.48456 (7)	0.0354 (3)	
C2	0.50350 (7)	0.73891 (11)	0.42227 (7)	0.0414 (3)	
H2A	0.533825	0.685033	0.439461	0.050*	
H2B	0.526999	0.774747	0.386800	0.050*	
C3	0.43522 (8)	0.69805 (11)	0.38527 (7)	0.0414 (3)	
H3A	0.401692	0.750812	0.375210	0.050*	
H3B	0.415691	0.652436	0.417694	0.050*	
C4	0.44582 (8)	0.64570 (10)	0.31390 (7)	0.0401 (3)	
H4A	0.483627	0.598510	0.322936	0.048*	
H4B	0.403179	0.610461	0.296658	0.048*	
C5	0.46305 (7)	0.71502 (10)	0.25702 (7)	0.0355 (3)	
C6	0.52320 (7)	0.80228 (10)	0.18440 (7)	0.0363 (3)	
C7	0.57440 (8)	0.84553 (12)	0.14697 (8)	0.0472 (3)	
H7	0.621668	0.828365	0.155748	0.057*	
C8	0.55114 (10)	0.91515 (12)	0.09623 (9)	0.0534 (4)	
H8	0.583527	0.945422	0.069696	0.064*	
C9	0.48050 (10)	0.94136 (12)	0.08362 (8)	0.0533 (4)	
H9	0.467233	0.989058	0.049086	0.064*	
C10	0.42940 (9)	0.89916 (11)	0.12051 (8)	0.0465 (3)	
H10	0.382221	0.916829	0.111873	0.056*	
C11	0.45284 (7)	0.82876 (10)	0.17126 (7)	0.0361 (3)	
C12	0.52597 (7)	0.91080 (10)	0.57163 (7)	0.0386 (3)	
C13	0.55887 (9)	0.97515 (13)	0.62200 (9)	0.0538 (4)	
H13	0.607032	0.987016	0.625226	0.065*	
C14	0.51623 (11)	1.02099 (13)	0.66740 (9)	0.0595 (4)	
H14	0.536547	1.064161	0.702378	0.071*	
C15	0.44410 (10)	1.00457 (12)	0.66236 (8)	0.0529 (4)	
H15	0.417313	1.037534	0.693492	0.063*	
C16	0.41135 (8)	0.94012 (11)	0.61192 (8)	0.0439 (3)	
H16	0.363096	0.928962	0.608597	0.053*	
C17	0.45356 (7)	0.89261 (10)	0.56630 (7)	0.0349 (3)	
N5	0.09472 (7)	0.38806 (10)	0.36023 (8)	0.0495 (3)	
H5	0.1403 (11)	0.3806 (14)	0.3680 (10)	0.059*	
N6	−0.01011 (7)	0.45637 (11)	0.36410 (8)	0.0521 (3)	
N7	0.10001 (7)	0.85178 (10)	0.56524 (7)	0.0456 (3)	
H7A	0.1453 (10)	0.8453 (13)	0.5791 (10)	0.055*	
N8	−0.00373 (7)	0.80897 (10)	0.51594 (8)	0.0483 (3)	
H8A	−0.0379 (10)	0.7772 (14)	0.4859 (10)	0.058*	
C18	0.06447 (8)	0.78607 (11)	0.52270 (8)	0.0455 (3)	
C19	0.09867 (9)	0.70444 (14)	0.48780 (12)	0.0624 (5)	
H19A	0.132165	0.674085	0.524166	0.075*	
H19B	0.125282	0.731627	0.450975	0.075*	
C20	0.05109 (8)	0.62524 (12)	0.45272 (9)	0.0492 (3)	
H20A	0.019172	0.652790	0.413636	0.059*	
H20B	0.023248	0.597311	0.488226	0.059*	
C21	0.09585 (10)	0.54661 (15)	0.42316 (14)	0.0730 (6)	
H21A	0.125815	0.577390	0.390850	0.088*	
H21B	0.126381	0.519348	0.463457	0.088*	
C22	0.05788 (8)	0.46473 (12)	0.38334 (9)	0.0488 (3)	
C23	0.04746 (8)	0.32482 (11)	0.32295 (8)	0.0448 (3)	
C24	0.05564 (9)	0.23748 (13)	0.28724 (10)	0.0566 (4)	
H24	0.099755	0.209510	0.285394	0.068*	
C25	−0.00474 (10)	0.19407 (14)	0.25462 (11)	0.0628 (4)	
H25	−0.001440	0.135152	0.230306	0.075*	
C26	−0.07055 (10)	0.23635 (15)	0.25720 (11)	0.0656 (5)	
H26	−0.110197	0.205101	0.234366	0.079*	
C27	−0.07857 (9)	0.32305 (15)	0.29257 (10)	0.0609 (4)	
H27	−0.122870	0.350593	0.294063	0.073*	
C28	−0.01816 (8)	0.36856 (12)	0.32624 (8)	0.0460 (3)	
C29	0.05361 (7)	0.92069 (11)	0.58757 (8)	0.0439 (3)	
C30	0.06356 (9)	1.00275 (13)	0.63086 (9)	0.0548 (4)	
H30	0.108105	1.021570	0.651519	0.066*	
C31	0.00397 (9)	1.05547 (14)	0.64194 (10)	0.0592 (4)	
H31	0.008415	1.111115	0.670836	0.071*	
C32	−0.06267 (9)	1.02692 (14)	0.61067 (11)	0.0597 (4)	
H32	−0.101573	1.063905	0.619671	0.072*	
C33	−0.07269 (8)	0.94611 (13)	0.56710 (10)	0.0559 (4)	
H33	−0.117224	0.927811	0.546088	0.067*	
C34	−0.01281 (8)	0.89285 (11)	0.55594 (8)	0.0447 (3)	
C35	0.27578 (7)	1.15743 (9)	0.54421 (6)	0.0327 (2)	
C36	0.26578 (6)	1.11098 (9)	0.47017 (6)	0.0311 (2)	
C37	0.26289 (7)	1.16953 (9)	0.40831 (7)	0.0347 (3)	
H37	0.269002	1.236684	0.412910	0.042*	
C38	0.25090 (7)	1.12754 (9)	0.33992 (7)	0.0347 (3)	
C39	0.24579 (7)	1.02643 (9)	0.33266 (7)	0.0349 (3)	
C40	0.24977 (7)	0.96838 (9)	0.39459 (7)	0.0353 (3)	
C41	0.25794 (7)	1.01032 (9)	0.46320 (7)	0.0341 (3)	
H41	0.258181	0.971322	0.504286	0.041*	
O1	0.32408 (6)	1.21949 (8)	0.55594 (6)	0.0507 (3)	
O2	0.23473 (6)	1.13157 (8)	0.58882 (5)	0.0456 (2)	
O3	0.24418 (7)	1.17940 (8)	0.27628 (5)	0.0503 (3)	
H3C	0.2437 (11)	1.2442 (18)	0.2827 (12)	0.075*	
O4	0.23704 (7)	0.98745 (8)	0.26442 (5)	0.0509 (3)	
H4C	0.2448 (11)	0.9203 (17)	0.2666 (12)	0.076*	
O5	0.24291 (7)	0.87063 (7)	0.38227 (6)	0.0525 (3)	
H5A	0.2628 (11)	0.8359 (17)	0.4219 (13)	0.079*	
C42	0.25350 (6)	0.71858 (8)	0.17515 (6)	0.0291 (2)	
C43	0.25385 (6)	0.63616 (8)	0.22838 (6)	0.0273 (2)	
C44	0.24764 (6)	0.54077 (8)	0.20308 (6)	0.0305 (2)	
H44	0.242484	0.528655	0.153415	0.037*	
C45	0.24910 (7)	0.46333 (8)	0.25170 (6)	0.0310 (2)	
C46	0.25561 (6)	0.48180 (8)	0.32612 (6)	0.0288 (2)	
C47	0.26286 (6)	0.57789 (8)	0.35153 (6)	0.0302 (2)	
C48	0.26155 (6)	0.65504 (8)	0.30281 (6)	0.0305 (2)	
H48	0.265776	0.718937	0.319694	0.037*	
O6	0.27500 (5)	0.80143 (6)	0.19854 (5)	0.0387 (2)	
O7	0.23446 (5)	0.70082 (7)	0.10908 (5)	0.0376 (2)	
O8	0.24400 (6)	0.36817 (6)	0.23073 (5)	0.0468 (3)	
H8B	0.2433 (11)	0.3648 (15)	0.1836 (13)	0.070*	
O9	0.25344 (5)	0.40454 (6)	0.37215 (5)	0.0369 (2)	
H9A	0.2689 (10)	0.4163 (14)	0.4144 (11)	0.055*	
O10	0.26956 (6)	0.58675 (7)	0.42477 (5)	0.0461 (3)	
H10A	0.2798 (11)	0.6453 (16)	0.4390 (11)	0.069*	
O11	0.23828 (10)	0.94991 (12)	0.08817 (8)	0.0787 (4)	
H11A	0.2465 (16)	0.906 (2)	0.1228 (18)	0.118*	
H11B	0.2023 (17)	0.987 (2)	0.1081 (17)	0.118*	
O12	0.64430 (7)	0.66889 (11)	0.31948 (7)	0.0594 (3)	
H12A	0.6632 (13)	0.7109 (18)	0.3574 (14)	0.089*	
H12B	0.6785 (14)	0.6568 (19)	0.2968 (14)	0.089*	
O13	0.29935 (5)	0.75961 (7)	0.48875 (5)	0.03507 (19)	
H13A	0.3455 (10)	0.7797 (13)	0.4988 (10)	0.053*	
H13B	0.2836 (9)	0.7643 (13)	0.5301 (10)	0.053*	
O14	0.31027 (8)	0.41737 (10)	0.50706 (6)	0.0626 (3)	
H14A	0.3083 (13)	0.357 (2)	0.5319 (14)	0.094*	
H14B	0.2845 (14)	0.461 (2)	0.5353 (14)	0.094*	
O15	−0.11852 (9)	0.73643 (15)	0.43842 (15)	0.1198 (9)	
H15A	−0.147 (2)	0.775 (3)	0.427 (2)	0.180*	
H15B	−0.128 (2)	0.681 (3)	0.418 (2)	0.180*	
O16	−0.13294 (8)	0.56909 (11)	0.35690 (9)	0.0709 (4)	
H16A	−0.1558 (15)	0.562 (2)	0.3116 (16)	0.106*	
H16B	−0.0854 (16)	0.536 (2)	0.3645 (15)	0.106*	

### 2-[3-(1H-Benzimidazol-2-yl)propyl]-1H-benzimidazol-3-ium 3,4,5-trihydroxybenzoate trihydrate Atomic displacement parameters (Å^2^)

**Table:**

	*U*^11^	*U*^22^	*U*^33^	*U*^12^	*U*^13^	*U*^23^
N1	0.0319 (5)	0.0501 (7)	0.0416 (6)	−0.0034 (5)	0.0066 (5)	−0.0024 (5)
N2	0.0371 (6)	0.0425 (6)	0.0326 (5)	−0.0037 (5)	0.0058 (4)	−0.0039 (4)
N3	0.0360 (6)	0.0433 (6)	0.0336 (5)	0.0031 (5)	0.0044 (4)	0.0000 (4)
N4	0.0338 (5)	0.0386 (6)	0.0379 (5)	−0.0027 (4)	0.0020 (4)	−0.0025 (4)
C1	0.0369 (6)	0.0383 (6)	0.0314 (6)	−0.0017 (5)	0.0050 (5)	0.0021 (5)
C2	0.0419 (7)	0.0472 (8)	0.0357 (6)	0.0017 (6)	0.0073 (5)	−0.0041 (6)
C3	0.0450 (7)	0.0451 (7)	0.0353 (6)	−0.0038 (6)	0.0103 (5)	−0.0035 (5)
C4	0.0480 (7)	0.0376 (7)	0.0357 (6)	−0.0034 (6)	0.0091 (5)	−0.0029 (5)
C5	0.0378 (6)	0.0369 (6)	0.0318 (6)	−0.0028 (5)	0.0038 (5)	−0.0073 (5)
C6	0.0407 (7)	0.0377 (6)	0.0309 (6)	−0.0007 (5)	0.0049 (5)	−0.0039 (5)
C7	0.0461 (8)	0.0524 (8)	0.0451 (8)	−0.0020 (6)	0.0140 (6)	−0.0008 (6)
C8	0.0675 (10)	0.0501 (9)	0.0458 (8)	−0.0057 (8)	0.0210 (7)	0.0031 (7)
C9	0.0748 (11)	0.0446 (8)	0.0405 (7)	−0.0003 (7)	0.0059 (7)	0.0060 (6)
C10	0.0518 (8)	0.0433 (7)	0.0424 (7)	0.0022 (6)	−0.0032 (6)	0.0008 (6)
C11	0.0397 (7)	0.0365 (6)	0.0315 (6)	−0.0025 (5)	0.0016 (5)	−0.0053 (5)
C12	0.0415 (7)	0.0392 (7)	0.0350 (6)	−0.0058 (5)	0.0038 (5)	0.0022 (5)
C13	0.0526 (9)	0.0544 (9)	0.0529 (9)	−0.0165 (7)	−0.0005 (7)	−0.0075 (7)
C14	0.0825 (12)	0.0467 (9)	0.0481 (9)	−0.0158 (8)	0.0018 (8)	−0.0122 (7)
C15	0.0768 (11)	0.0429 (8)	0.0408 (7)	0.0014 (7)	0.0151 (7)	−0.0046 (6)
C16	0.0483 (8)	0.0458 (8)	0.0393 (7)	0.0008 (6)	0.0119 (6)	−0.0011 (6)
C17	0.0396 (6)	0.0354 (6)	0.0297 (5)	−0.0029 (5)	0.0045 (5)	0.0013 (5)
N5	0.0361 (6)	0.0516 (7)	0.0598 (8)	0.0016 (5)	0.0005 (5)	−0.0060 (6)
N6	0.0370 (6)	0.0601 (8)	0.0593 (8)	0.0018 (6)	0.0062 (5)	−0.0065 (6)
N7	0.0347 (6)	0.0530 (7)	0.0470 (7)	0.0068 (5)	−0.0043 (5)	−0.0031 (5)
N8	0.0349 (6)	0.0508 (7)	0.0576 (8)	0.0022 (5)	−0.0021 (5)	−0.0050 (6)
C18	0.0387 (7)	0.0486 (8)	0.0479 (8)	0.0040 (6)	−0.0006 (6)	−0.0005 (6)
C19	0.0428 (8)	0.0587 (10)	0.0837 (13)	0.0053 (7)	−0.0021 (8)	−0.0204 (9)
C20	0.0428 (8)	0.0547 (9)	0.0499 (8)	−0.0002 (7)	0.0046 (6)	−0.0064 (7)
C21	0.0437 (9)	0.0670 (12)	0.1053 (16)	0.0046 (8)	−0.0051 (9)	−0.0332 (11)
C22	0.0392 (7)	0.0531 (9)	0.0538 (8)	0.0011 (6)	0.0038 (6)	−0.0050 (7)
C23	0.0411 (7)	0.0486 (8)	0.0444 (7)	−0.0036 (6)	0.0037 (6)	0.0028 (6)
C24	0.0540 (9)	0.0527 (9)	0.0630 (10)	0.0015 (7)	0.0057 (8)	−0.0018 (8)
C25	0.0681 (11)	0.0561 (10)	0.0645 (10)	−0.0119 (9)	0.0085 (9)	−0.0095 (8)
C26	0.0567 (10)	0.0724 (12)	0.0672 (11)	−0.0215 (9)	0.0044 (8)	−0.0088 (9)
C27	0.0401 (8)	0.0767 (12)	0.0661 (11)	−0.0092 (8)	0.0072 (7)	−0.0058 (9)
C28	0.0393 (7)	0.0545 (9)	0.0445 (7)	−0.0052 (6)	0.0064 (6)	0.0020 (6)
C29	0.0386 (7)	0.0526 (8)	0.0399 (7)	0.0068 (6)	0.0012 (5)	0.0013 (6)
C30	0.0464 (8)	0.0643 (10)	0.0519 (9)	0.0023 (7)	−0.0023 (7)	−0.0102 (8)
C31	0.0571 (10)	0.0621 (10)	0.0589 (10)	0.0063 (8)	0.0080 (8)	−0.0122 (8)
C32	0.0483 (9)	0.0616 (10)	0.0710 (11)	0.0117 (8)	0.0153 (8)	−0.0025 (9)
C33	0.0361 (7)	0.0602 (10)	0.0715 (11)	0.0057 (7)	0.0062 (7)	−0.0013 (8)
C34	0.0379 (7)	0.0486 (8)	0.0471 (8)	0.0030 (6)	0.0029 (6)	0.0018 (6)
C35	0.0389 (6)	0.0273 (5)	0.0310 (5)	0.0043 (5)	0.0007 (5)	0.0023 (4)
C36	0.0330 (6)	0.0290 (5)	0.0311 (5)	0.0014 (4)	0.0029 (4)	0.0011 (4)
C37	0.0420 (7)	0.0262 (5)	0.0361 (6)	−0.0002 (5)	0.0061 (5)	0.0032 (5)
C38	0.0419 (7)	0.0310 (6)	0.0315 (6)	0.0025 (5)	0.0057 (5)	0.0070 (5)
C39	0.0408 (6)	0.0325 (6)	0.0305 (6)	0.0023 (5)	0.0002 (5)	0.0015 (5)
C40	0.0441 (7)	0.0264 (6)	0.0344 (6)	0.0013 (5)	−0.0003 (5)	0.0013 (4)
C41	0.0435 (7)	0.0279 (6)	0.0304 (6)	0.0005 (5)	0.0015 (5)	0.0047 (4)
O1	0.0561 (6)	0.0516 (6)	0.0435 (5)	−0.0157 (5)	0.0018 (5)	−0.0086 (5)
O2	0.0578 (6)	0.0472 (6)	0.0334 (5)	−0.0042 (5)	0.0114 (4)	0.0003 (4)
O3	0.0840 (8)	0.0333 (5)	0.0340 (5)	0.0042 (5)	0.0084 (5)	0.0103 (4)
O4	0.0819 (8)	0.0373 (5)	0.0315 (5)	0.0053 (5)	−0.0029 (5)	−0.0017 (4)
O5	0.0904 (8)	0.0260 (5)	0.0381 (5)	0.0017 (5)	−0.0065 (5)	−0.0002 (4)
C42	0.0245 (5)	0.0295 (5)	0.0332 (5)	−0.0008 (4)	0.0024 (4)	0.0047 (4)
C43	0.0284 (5)	0.0253 (5)	0.0282 (5)	−0.0012 (4)	0.0027 (4)	0.0022 (4)
C44	0.0369 (6)	0.0287 (5)	0.0260 (5)	−0.0013 (4)	0.0040 (4)	−0.0006 (4)
C45	0.0393 (6)	0.0232 (5)	0.0309 (5)	−0.0006 (4)	0.0048 (4)	−0.0020 (4)
C46	0.0335 (6)	0.0247 (5)	0.0282 (5)	−0.0007 (4)	0.0043 (4)	0.0023 (4)
C47	0.0371 (6)	0.0277 (5)	0.0259 (5)	−0.0030 (4)	0.0044 (4)	−0.0013 (4)
C48	0.0382 (6)	0.0236 (5)	0.0297 (5)	−0.0029 (4)	0.0031 (4)	−0.0014 (4)
O6	0.0449 (5)	0.0267 (4)	0.0435 (5)	−0.0046 (4)	0.0004 (4)	0.0055 (4)
O7	0.0393 (5)	0.0422 (5)	0.0307 (4)	−0.0077 (4)	0.0011 (3)	0.0071 (4)
O8	0.0875 (8)	0.0230 (4)	0.0298 (4)	−0.0028 (4)	0.0067 (5)	−0.0031 (3)
O9	0.0537 (6)	0.0272 (4)	0.0295 (4)	−0.0034 (4)	0.0033 (4)	0.0043 (3)
O10	0.0817 (8)	0.0313 (5)	0.0252 (4)	−0.0098 (5)	0.0055 (4)	−0.0021 (3)
O11	0.1061 (12)	0.0686 (9)	0.0664 (9)	0.0174 (8)	0.0319 (8)	0.0241 (7)
O12	0.0491 (7)	0.0839 (9)	0.0438 (6)	0.0090 (6)	−0.0017 (5)	−0.0066 (6)
O13	0.0400 (5)	0.0350 (5)	0.0302 (4)	−0.0022 (4)	0.0038 (4)	−0.0040 (3)
O14	0.0956 (10)	0.0511 (7)	0.0407 (6)	0.0071 (7)	0.0048 (6)	0.0052 (5)
O15	0.0562 (9)	0.0945 (13)	0.196 (2)	0.0233 (8)	−0.0446 (11)	−0.0667 (14)
O16	0.0526 (7)	0.0784 (9)	0.0787 (9)	0.0016 (6)	−0.0064 (6)	−0.0155 (7)

### 2-[3-(1H-Benzimidazol-2-yl)propyl]-1H-benzimidazol-3-ium 3,4,5-trihydroxybenzoate trihydrate Geometric parameters (Å, º)

**Table:**

N1—C1	1.3514 (17)	C24—C25	1.377 (3)
N1—C12	1.3766 (18)	C24—H24	0.9300
N1—H1	0.818 (18)	C25—C26	1.390 (3)
N2—C1	1.3162 (17)	C25—H25	0.9300
N2—C17	1.3942 (16)	C26—C27	1.373 (3)
N3—C5	1.3304 (17)	C26—H26	0.9300
N3—C6	1.3852 (17)	C27—C28	1.397 (2)
N3—H3	0.885 (17)	C27—H27	0.9300
N4—C5	1.3344 (17)	C29—C30	1.382 (2)
N4—C11	1.3890 (17)	C29—C34	1.391 (2)
N4—H4	0.878 (17)	C30—C31	1.383 (2)
C1—C2	1.4894 (18)	C30—H30	0.9300
C2—C3	1.512 (2)	C31—C32	1.395 (3)
C2—H2A	0.9700	C31—H31	0.9300
C2—H2B	0.9700	C32—C33	1.371 (3)
C3—C4	1.5381 (18)	C32—H32	0.9300
C3—H3A	0.9700	C33—C34	1.392 (2)
C3—H3B	0.9700	C33—H33	0.9300
C4—C5	1.4832 (19)	C35—O2	1.2528 (16)
C4—H4A	0.9700	C35—O1	1.2558 (16)
C4—H4B	0.9700	C35—C36	1.5057 (17)
C6—C11	1.3876 (19)	C36—C41	1.3914 (17)
C6—C7	1.393 (2)	C36—C37	1.3955 (17)
C7—C8	1.378 (2)	C37—C38	1.3879 (18)
C7—H7	0.9300	C37—H37	0.9300
C8—C9	1.391 (3)	C38—O3	1.3708 (15)
C8—H8	0.9300	C38—C39	1.3939 (18)
C9—C10	1.380 (2)	C39—O4	1.3662 (15)
C9—H9	0.9300	C39—C40	1.3917 (17)
C10—C11	1.386 (2)	C40—O5	1.3621 (15)
C10—H10	0.9300	C40—C41	1.3886 (17)
C12—C13	1.383 (2)	C41—H41	0.9300
C12—C17	1.3987 (18)	O3—H3C	0.90 (2)
C13—C14	1.385 (3)	O4—H4C	0.93 (2)
C13—H13	0.9300	O5—H5A	0.92 (2)
C14—C15	1.389 (3)	C42—O7	1.2621 (15)
C14—H14	0.9300	C42—O6	1.2668 (15)
C15—C16	1.384 (2)	C42—C43	1.4990 (15)
C15—H15	0.9300	C43—C44	1.3888 (16)
C16—C17	1.3934 (19)	C43—C48	1.3956 (16)
C16—H16	0.9300	C44—C45	1.3903 (16)
N5—C22	1.360 (2)	C44—H44	0.9300
N5—C23	1.380 (2)	C45—O8	1.3606 (14)
N5—H5	0.87 (2)	C45—C46	1.3947 (16)
N6—C22	1.3135 (19)	C46—O9	1.3630 (14)
N6—C28	1.392 (2)	C46—C47	1.3996 (16)
N7—C18	1.332 (2)	C47—O10	1.3550 (14)
N7—C29	1.3895 (19)	C47—C48	1.3885 (16)
N7—H7A	0.879 (19)	C48—H48	0.9300
N8—C18	1.3326 (19)	O8—H8B	0.87 (2)
N8—C34	1.389 (2)	O9—H9A	0.82 (2)
N8—H8A	0.919 (19)	O10—H10A	0.86 (2)
C18—C19	1.480 (2)	O11—H11A	0.88 (3)
C19—C20	1.515 (2)	O11—H11B	0.96 (3)
C19—H19A	0.9700	O12—H12A	0.95 (3)
C19—H19B	0.9700	O12—H12B	0.83 (3)
C20—C21	1.515 (2)	O13—H13A	0.923 (19)
C20—H20A	0.9700	O13—H13B	0.856 (19)
C20—H20B	0.9700	O14—H14A	0.94 (3)
C21—C22	1.487 (2)	O14—H14B	0.97 (3)
C21—H21A	0.9700	O15—H15A	0.77 (5)
C21—H21B	0.9700	O15—H15B	0.86 (5)
C23—C24	1.385 (2)	O16—H16A	0.91 (3)
C23—C28	1.397 (2)	O16—H16B	1.01 (3)
			
C1—N1—C12	107.86 (11)	C20—C21—H21A	108.1
C1—N1—H1	125.6 (13)	C22—C21—H21B	108.1
C12—N1—H1	126.4 (13)	C20—C21—H21B	108.1
C1—N2—C17	105.01 (11)	H21A—C21—H21B	107.3
C5—N3—C6	109.30 (12)	N6—C22—N5	112.22 (14)
C5—N3—H3	125.9 (11)	N6—C22—C21	127.87 (15)
C6—N3—H3	124.7 (11)	N5—C22—C21	119.87 (14)
C5—N4—C11	109.12 (11)	N5—C23—C24	132.79 (15)
C5—N4—H4	125.5 (11)	N5—C23—C28	104.44 (14)
C11—N4—H4	125.3 (11)	C24—C23—C28	122.76 (14)
N2—C1—N1	112.64 (12)	C25—C24—C23	116.70 (16)
N2—C1—C2	126.63 (12)	C25—C24—H24	121.6
N1—C1—C2	120.70 (12)	C23—C24—H24	121.6
C1—C2—C3	114.09 (11)	C24—C25—C26	121.48 (18)
C1—C2—H2A	108.7	C24—C25—H25	119.3
C3—C2—H2A	108.7	C26—C25—H25	119.3
C1—C2—H2B	108.7	C27—C26—C25	121.75 (17)
C3—C2—H2B	108.7	C27—C26—H26	119.1
H2A—C2—H2B	107.6	C25—C26—H26	119.1
C2—C3—C4	111.90 (11)	C26—C27—C28	117.95 (17)
C2—C3—H3A	109.2	C26—C27—H27	121.0
C4—C3—H3A	109.2	C28—C27—H27	121.0
C2—C3—H3B	109.2	N6—C28—C27	130.56 (15)
C4—C3—H3B	109.2	N6—C28—C23	110.07 (13)
H3A—C3—H3B	107.9	C27—C28—C23	119.36 (16)
C5—C4—C3	111.97 (11)	C30—C29—N7	132.42 (14)
C5—C4—H4A	109.2	C30—C29—C34	121.87 (14)
C3—C4—H4A	109.2	N7—C29—C34	105.71 (13)
C5—C4—H4B	109.2	C29—C30—C31	116.66 (15)
C3—C4—H4B	109.2	C29—C30—H30	121.7
H4A—C4—H4B	107.9	C31—C30—H30	121.7
N3—C5—N4	108.90 (12)	C30—C31—C32	121.36 (17)
N3—C5—C4	125.47 (12)	C30—C31—H31	119.3
N4—C5—C4	125.61 (12)	C32—C31—H31	119.3
N3—C6—C11	106.44 (11)	C33—C32—C31	122.16 (16)
N3—C6—C7	132.10 (13)	C33—C32—H32	118.9
C11—C6—C7	121.46 (13)	C31—C32—H32	118.9
C8—C7—C6	116.32 (15)	C32—C33—C34	116.59 (15)
C8—C7—H7	121.8	C32—C33—H33	121.7
C6—C7—H7	121.8	C34—C33—H33	121.7
C7—C8—C9	121.76 (15)	N8—C34—C29	106.83 (13)
C7—C8—H8	119.1	N8—C34—C33	131.82 (15)
C9—C8—H8	119.1	C29—C34—C33	121.35 (15)
C10—C9—C8	122.38 (15)	O2—C35—O1	125.40 (12)
C10—C9—H9	118.8	O2—C35—C36	117.21 (11)
C8—C9—H9	118.8	O1—C35—C36	117.39 (11)
C9—C10—C11	115.77 (14)	C41—C36—C37	119.94 (11)
C9—C10—H10	122.1	C41—C36—C35	120.32 (11)
C11—C10—H10	122.1	C37—C36—C35	119.72 (11)
C10—C11—C6	122.31 (13)	C38—C37—C36	120.01 (11)
C10—C11—N4	131.45 (13)	C38—C37—H37	120.0
C6—C11—N4	106.24 (11)	C36—C37—H37	120.0
N1—C12—C13	132.94 (14)	O3—C38—C37	124.17 (11)
N1—C12—C17	104.87 (11)	O3—C38—C39	115.65 (11)
C13—C12—C17	122.20 (14)	C37—C38—C39	120.17 (11)
C12—C13—C14	116.43 (15)	O4—C39—C40	122.04 (12)
C12—C13—H13	121.8	O4—C39—C38	118.54 (11)
C14—C13—H13	121.8	C40—C39—C38	119.42 (11)
C13—C14—C15	122.15 (15)	O5—C40—C41	123.99 (11)
C13—C14—H14	118.9	O5—C40—C39	115.34 (11)
C15—C14—H14	118.9	C41—C40—C39	120.64 (11)
C16—C15—C14	121.27 (15)	C40—C41—C36	119.67 (11)
C16—C15—H15	119.4	C40—C41—H41	120.2
C14—C15—H15	119.4	C36—C41—H41	120.2
C15—C16—C17	117.35 (14)	C38—O3—H3C	113.6 (14)
C15—C16—H16	121.3	C39—O4—H4C	110.0 (14)
C17—C16—H16	121.3	C40—O5—H5A	110.5 (14)
C16—C17—N2	129.79 (13)	O7—C42—O6	123.53 (11)
C16—C17—C12	120.59 (13)	O7—C42—C43	118.25 (10)
N2—C17—C12	109.61 (11)	O6—C42—C43	118.15 (10)
C22—N5—C23	108.04 (13)	C44—C43—C48	120.26 (10)
C22—N5—H5	125.5 (13)	C44—C43—C42	119.49 (10)
C23—N5—H5	126.5 (13)	C48—C43—C42	120.24 (10)
C22—N6—C28	105.22 (13)	C43—C44—C45	120.27 (11)
C18—N7—C29	109.59 (12)	C43—C44—H44	119.9
C18—N7—H7A	121.7 (12)	C45—C44—H44	119.9
C29—N7—H7A	128.4 (12)	O8—C45—C44	123.37 (11)
C18—N8—C34	108.88 (13)	O8—C45—C46	116.88 (10)
C18—N8—H8A	124.5 (12)	C44—C45—C46	119.74 (10)
C34—N8—H8A	126.3 (12)	O9—C46—C45	118.23 (10)
N7—C18—N8	108.99 (13)	O9—C46—C47	121.88 (10)
N7—C18—C19	123.26 (14)	C45—C46—C47	119.88 (10)
N8—C18—C19	127.71 (14)	O10—C47—C48	125.21 (11)
C18—C19—C20	117.10 (14)	O10—C47—C46	114.60 (10)
C18—C19—H19A	108.0	C48—C47—C46	120.19 (10)
C20—C19—H19A	108.0	C47—C48—C43	119.63 (10)
C18—C19—H19B	108.0	C47—C48—H48	120.2
C20—C19—H19B	108.0	C43—C48—H48	120.2
H19A—C19—H19B	107.3	C45—O8—H8B	109.2 (14)
C19—C20—C21	109.17 (13)	C46—O9—H9A	114.3 (13)
C19—C20—H20A	109.8	C47—O10—H10A	112.5 (14)
C21—C20—H20A	109.8	H11A—O11—H11B	99 (2)
C19—C20—H20B	109.8	H12A—O12—H12B	104 (2)
C21—C20—H20B	109.8	H13A—O13—H13B	102.7 (16)
H20A—C20—H20B	108.3	H14A—O14—H14B	103 (2)
C22—C21—C20	116.86 (15)	H15A—O15—H15B	112 (4)
C22—C21—H21A	108.1	H16A—O16—H16B	114 (2)
			
C17—N2—C1—N1	0.55 (15)	C25—C26—C27—C28	0.2 (3)
C17—N2—C1—C2	−177.43 (13)	C22—N6—C28—C27	−179.17 (18)
C12—N1—C1—N2	−0.45 (16)	C22—N6—C28—C23	−0.45 (18)
C12—N1—C1—C2	177.66 (12)	C26—C27—C28—N6	178.48 (17)
N2—C1—C2—C3	3.7 (2)	C26—C27—C28—C23	−0.1 (3)
N1—C1—C2—C3	−174.12 (13)	N5—C23—C28—N6	0.52 (17)
C1—C2—C3—C4	170.03 (12)	C24—C23—C28—N6	−178.67 (15)
C2—C3—C4—C5	−69.39 (16)	N5—C23—C28—C27	179.40 (15)
C6—N3—C5—N4	−0.36 (14)	C24—C23—C28—C27	0.2 (2)
C6—N3—C5—C4	−178.65 (12)	C18—N7—C29—C30	−179.43 (17)
C11—N4—C5—N3	0.53 (14)	C18—N7—C29—C34	0.05 (17)
C11—N4—C5—C4	178.82 (12)	N7—C29—C30—C31	−179.88 (17)
C3—C4—C5—N3	101.02 (15)	C34—C29—C30—C31	0.7 (2)
C3—C4—C5—N4	−76.99 (16)	C29—C30—C31—C32	−0.1 (3)
C5—N3—C6—C11	0.06 (14)	C30—C31—C32—C33	−0.5 (3)
C5—N3—C6—C7	179.51 (14)	C31—C32—C33—C34	0.6 (3)
N3—C6—C7—C8	−179.78 (14)	C18—N8—C34—C29	0.21 (18)
C11—C6—C7—C8	−0.4 (2)	C18—N8—C34—C33	−179.73 (17)
C6—C7—C8—C9	0.6 (2)	C30—C29—C34—N8	179.40 (15)
C7—C8—C9—C10	−0.5 (3)	N7—C29—C34—N8	−0.16 (17)
C8—C9—C10—C11	0.1 (2)	C30—C29—C34—C33	−0.7 (2)
C9—C10—C11—C6	0.1 (2)	N7—C29—C34—C33	179.79 (15)
C9—C10—C11—N4	179.24 (14)	C32—C33—C34—N8	179.93 (17)
N3—C6—C11—C10	179.59 (12)	C32—C33—C34—C29	0.0 (3)
C7—C6—C11—C10	0.1 (2)	O2—C35—C36—C41	−47.51 (17)
N3—C6—C11—N4	0.26 (14)	O1—C35—C36—C41	133.14 (13)
C7—C6—C11—N4	−179.27 (12)	O2—C35—C36—C37	131.13 (13)
C5—N4—C11—C10	−179.73 (14)	O1—C35—C36—C37	−48.22 (17)
C5—N4—C11—C6	−0.49 (14)	C41—C36—C37—C38	1.20 (19)
C1—N1—C12—C13	−179.66 (16)	C35—C36—C37—C38	−177.45 (12)
C1—N1—C12—C17	0.14 (15)	C36—C37—C38—O3	177.07 (13)
N1—C12—C13—C14	179.88 (16)	C36—C37—C38—C39	−3.7 (2)
C17—C12—C13—C14	0.1 (2)	O3—C38—C39—O4	2.09 (19)
C12—C13—C14—C15	−0.8 (3)	C37—C38—C39—O4	−177.18 (12)
C13—C14—C15—C16	0.8 (3)	O3—C38—C39—C40	−177.97 (12)
C14—C15—C16—C17	−0.2 (2)	C37—C38—C39—C40	2.8 (2)
C15—C16—C17—N2	−179.85 (14)	O4—C39—C40—O5	−1.3 (2)
C15—C16—C17—C12	−0.5 (2)	C38—C39—C40—O5	178.80 (13)
C1—N2—C17—C16	178.96 (14)	O4—C39—C40—C41	−179.33 (13)
C1—N2—C17—C12	−0.45 (14)	C38—C39—C40—C41	0.7 (2)
N1—C12—C17—C16	−179.28 (13)	O5—C40—C41—C36	178.87 (13)
C13—C12—C17—C16	0.5 (2)	C39—C40—C41—C36	−3.2 (2)
N1—C12—C17—N2	0.18 (14)	C37—C36—C41—C40	2.27 (19)
C13—C12—C17—N2	−179.98 (14)	C35—C36—C41—C40	−179.09 (12)
C29—N7—C18—N8	0.08 (18)	O7—C42—C43—C44	13.10 (16)
C29—N7—C18—C19	177.95 (16)	O6—C42—C43—C44	−164.16 (11)
C34—N8—C18—N7	−0.18 (18)	O7—C42—C43—C48	−167.98 (11)
C34—N8—C18—C19	−177.93 (17)	O6—C42—C43—C48	14.77 (17)
N7—C18—C19—C20	168.86 (16)	C48—C43—C44—C45	−0.05 (18)
N8—C18—C19—C20	−13.7 (3)	C42—C43—C44—C45	178.87 (11)
C18—C19—C20—C21	−177.53 (18)	C43—C44—C45—O8	−179.34 (12)
C19—C20—C21—C22	−177.07 (19)	C43—C44—C45—C46	1.12 (18)
C28—N6—C22—N5	0.20 (19)	O8—C45—C46—O9	−2.70 (17)
C28—N6—C22—C21	177.97 (19)	C44—C45—C46—O9	176.87 (11)
C23—N5—C22—N6	0.1 (2)	O8—C45—C46—C47	178.41 (11)
C23—N5—C22—C21	−177.85 (17)	C44—C45—C46—C47	−2.02 (18)
C20—C21—C22—N6	7.4 (3)	O9—C46—C47—O10	1.88 (17)
C20—C21—C22—N5	−175.00 (17)	C45—C46—C47—O10	−179.27 (11)
C22—N5—C23—C24	178.69 (18)	O9—C46—C47—C48	−176.98 (11)
C22—N5—C23—C28	−0.39 (17)	C45—C46—C47—C48	1.87 (18)
N5—C23—C24—C25	−179.23 (17)	O10—C47—C48—C43	−179.53 (12)
C28—C23—C24—C25	−0.3 (3)	C46—C47—C48—C43	−0.81 (18)
C23—C24—C25—C26	0.3 (3)	C44—C43—C48—C47	−0.10 (18)
C24—C25—C26—C27	−0.3 (3)	C42—C43—C48—C47	−179.02 (11)

### 2-[3-(1H-Benzimidazol-2-yl)propyl]-1H-benzimidazol-3-ium 3,4,5-trihydroxybenzoate trihydrate Hydrogen-bond geometry (Å, º)

**Table:**

*D*—H···*A*	*D*—H	H···*A*	*D*···*A*	*D*—H···*A*
N1—H1···O1^i^	0.818 (18)	2.330 (18)	3.0706 (17)	150.9 (16)
N3—H3···O12	0.885 (17)	1.830 (18)	2.6968 (17)	166.0 (16)
N4—H4···O6	0.878 (17)	1.884 (18)	2.7290 (15)	160.9 (16)
N5—H5···O9	0.87 (2)	2.18 (2)	3.0248 (17)	162.9 (18)
N7—H7*A*···O7^ii^	0.879 (19)	1.84 (2)	2.7045 (15)	165.7 (18)
N8—H8*A*···O15	0.919 (19)	1.78 (2)	2.676 (2)	165.3 (18)
O3—H3*C*···O8^iii^	0.90 (2)	1.95 (2)	2.7201 (14)	142.6 (19)
O4—H4*C*···O6	0.93 (2)	2.18 (2)	2.9509 (14)	139.8 (19)
O5—H5*A*···O13	0.92 (2)	1.71 (2)	2.6263 (14)	173 (2)
O8—H8*B*···O2^iv^	0.87 (2)	1.75 (2)	2.6171 (13)	174 (2)
O9—H9*A*···O14	0.82 (2)	1.81 (2)	2.6193 (15)	168.4 (19)
O10—H10*A*···O13	0.86 (2)	1.83 (2)	2.6808 (13)	168 (2)
O11—H11*A*···O6	0.88 (3)	2.04 (3)	2.9148 (17)	174 (3)
O11—H11*B*···O16^v^	0.96 (3)	1.91 (3)	2.865 (2)	175 (3)
O12—H12*A*···O1^i^	0.95 (3)	1.86 (3)	2.7796 (17)	162 (2)
O12—H12*B*···O3^vi^	0.83 (3)	2.14 (3)	2.9254 (18)	158 (2)
O13—H13*A*···N2	0.923 (19)	1.81 (2)	2.7348 (15)	175.0 (17)
O13—H13*B*···O7^ii^	0.856 (19)	1.89 (2)	2.7249 (13)	165.5 (18)
O14—H14*A*···O1^vii^	0.94 (3)	1.96 (3)	2.8608 (18)	160 (2)
O14—H14*B*···O11^ii^	0.97 (3)	1.85 (3)	2.814 (2)	177 (2)
O15—H15*A*···O2^viii^	0.77 (5)	2.10 (5)	2.865 (2)	171 (5)
O15—H15*B*···O16	0.86 (5)	1.91 (5)	2.742 (2)	165 (4)
O16—H16*A*···O4^ix^	0.91 (3)	2.22 (3)	3.0444 (18)	150 (2)
O16—H16*B*···N6	1.01 (3)	1.80 (3)	2.800 (2)	167 (2)

Symmetry codes: (i) −*x*+1, −*y*+2, −*z*+1; (ii) *x*, −*y*+3/2, *z*+1/2; (iii) *x*, *y*+1, *z*; (iv) *x*, −*y*+3/2, *z*−1/2; (v) −*x*, *y*+1/2, −*z*+1/2; (vi) −*x*+1, *y*−1/2, −*z*+1/2; (vii) *x*, *y*−1, *z*; (viii) −*x*, −*y*+2, −*z*+1; (ix) −*x*, *y*−1/2, −*z*+1/2.
